# Hydrothermal growth of VO_2_ nanoplate thermochromic films on glass with high visible transmittance

**DOI:** 10.1038/srep27898

**Published:** 2016-06-14

**Authors:** Jiasong Zhang, Jingbo Li, Pengwan Chen, Fida Rehman, Yijie Jiang, Maosheng Cao, Yongjie Zhao, Haibo Jin

**Affiliations:** 1 Beijing Key Laboratory of Construction Tailorable Advanced Functional Materials and Green Applications, School of Materials Science and Engineering, Beijing Institute of Technology, Beijing 100081, China; 2 Department of Mechanical Engineering and Applied Mechanics, University of Pennsylvania, Philadelphia, Pennsylvania 19104, USA

## Abstract

The preparation of thermochromic vanadium dioxide (VO_2_) films in an economical way is of interest to realizing the application of smart windows. Here, we reported a successful preparation of self-assembly VO_2_ nanoplate films on TiO_2_-buffered glass by a facile hydrothermal process. The VO_2_ films composed of triangle-shaped plates standing on substrates exhibit a self-generated porous structure, which favors the transmission of solar light. The porosity of films is easily controlled by changing the concentration of precursor solutions. Excellent thermochromic properties are observed with visible light transmittance as high as 70.3% and solar modulating efficiency up to 9.3% in a VO_2_ film with porosity of ~35.9%. This work demonstrates a promising technique to promote the commercial utilization of VO_2_ in smart windows.

Energy consumption in the residential, commercial and other man-made buildings accounts for nearly 40% of total global energy use, making it the largest single component of energy use^1^. The explosion in demand for air-conditioning units is aggravating this large energy consumption. Low-E window which exhibits high reflectivity of infrared light has been widely used in commercial buildings to achieve energy saving. However, its solar radiation reflection has no responding ability to environmental temperature change, limiting its application in different market requirements^1^,^2^. Smart windows with thermochromic thin-film coatings on building glass provide an effective way to modulate the solar energy transmitted into the interior room.

It is well known that vanadium dioxide (VO_2_) shows a reversible metal-insulator phase transition (MIT) at a phase-transition temperature (*T*_c_) of 68 °C^3^. When temperature below *T*_c_, VO_2_ is an insulator with a monoclinic structure (*M* phase, space group *P*2_1_/c) which is transparent to infrared radiation (IR). As temperature above *T*_c_, VO_2_ transforms to a metallic state with a rutile structure (*R* phase, space group *P*4_2_/mnm) which is reflective to IR radiation while maintains visible-light transparent^4^. Such a MIT transition makes VO_2_ an attractive material for smart windows^5^. In order to promote the application of VO_2_ based smart windows, various methods have been used to achieve the VO_2_ coatings on transparent substrates for smart windows. However, how to enhance the visible light transmission with little sacrifice of solar modulation ability and lower the cost of large scale coating are still two major challenges for researchers. Based on the vapor-based deposition techniques^6^,^7^, multilayered structure (glass/TiO_2_/VO_2_/TiO_2_/VO_2_/TiO_2_)^8^, multifunctional TiO_2_(*R*)/VO_2_(*M*)/TiO_2_(*A*)^9^ and antireflection (AR) coatings on VO_2_ films^10^, were designed to meet the performance boost. But those methods were proved to be complicated and expensive due to the difficulties in controlling variable valences of V ions and costly equipment.

Recently, solution-based methods for depositing VO_2_ coatings on substrates have been studied extensively because of its low-cost and up-scalable. Cao *et al.* and Kang *et al.* have utilized solution methods (dip-coated with freeze-drying and spin-coated with sol-gel, respectively^11^,^12^) to obtain enhanced optical performance with high solar modulation ability. Their work demonstrated that creating tunable porosity in VO_2_ films was a feasible way to meet the performance requirements for practical usage. However, complex processes with high temperature crystallization treatment (500–550 °C for hours) were needed during those fabrications to limit their usability in industry.

Compared to those traditional solution-based deposition methods, hydrothermal method shows many advantages, such as easy implementation on the industrial scale, controllable porosity and crystal size, low-temperature processing, possibility to utilize a wide range of substrates, and being environmentally friendly. The hydrothermal technique has been used to grow ZnO films^13^, TiO_2_ films^14^ and other transition metal oxide functional thin films^15^,^16^ on glass or conductive substrate with high quality. Crystal morphologies, especially tunable porosity of films can be controlled by synthesis processes, showing great impacts on functional performance^13^. In previous studies, hydrothermal technique and subsequent thermal treatment were used to synthesize various VO_2_ (*M*) nanomaterials^17^,^18^, and VO_2_-based composite membrane were prepared by mixing VO_2_ (*M*) nanopowders with transparent polymer (e.g.,VO_2_/SiO_2_ core-shell^19^, VO_2_/ATO/polymer^20^ and polymer-assisted deposition^21^,^22^,^23^,^24^). However, there is no report about using the hydrothermal method to prepare VO_2_ (*M*) thin films on glass for smart windows.

To our knowledge, preparing a high quality metallic oxide thin film directly on glass by hydrothermal method is not easy^25^. The substrates with polarity and crystal orientations were usually used to grow fine organized thin films^26^,^27^,^28^. Our recent work has demonstrated that high quality epitaxial VO_2_ thin films can be grown on sapphire substrates by hydrothermal method^29^. Compared to the costly single crystal substrate, the buffer layer prepared on glass is an economic way to grow fine films. For example, Podlogar *et al.* prepared ZnO buffer layers on glass to grow highly adhesive crystalline ZnO films^13^, and Masuda *et al.* grew super hydrophilic TiO_2_ thin films on glass with SnO_2_:F layer (FTO)^30^.

Here, we successfully prepared VO_2_ smart windows via a facile hydrothermal process followed by a short heat treatment. High quality and porosity of obtained VO_2_ coatings make the films exhibit excellent thermochromic properties with good solar modulation ability and high visible light transmittance. To grow VO_2_ thin films on glass, TiO_2_ was selected as an buffer layer since TiO_2_ film shows stable thermal properties, high transparency to visible light and easy preparation^9^,^31^. The porosity of VO_2_ films was easily controlled by adjusting the concentration of the reaction solution. The possible growth mechanism was discussed based on the investigation into the effects of pH value and different precursor solutions on the growth process. The proposed simple process which is low cost and up-scalable would promote the application of VO_2_ in smart windows.

## Experimental

### Experiment section

All reagents used in the experiment were analytically pure and purchased from Sinopharm Chemical Reagent Co., Ltd. Vanadyl oxalate aqueous solution was used to grow VO_2_ thin films on glass substrates by the hydrothermal method. Before the growth of VO_2_ films, TiO_2_ buffers were firstly deposited on amorphous glass substrate by spin coating. A moderate-temperature treatment (400 °C) was carried out to achieve its crystallization and adhesion^32^. The detailed preparation process for TiO_2_ buffers is as follows: firstly, tetrabutyltitanate (C_16_H_36_O_4_Ti, 10 ml) was added into the ethanol (5 ml) at room temperature and stirred for 30 min. Then the solution was transferred into a mixed solution of nitric acid (3 ml), deionized water (6 ml) and ethanol (80 ml) and stirred for 1 h. Finally a transparent and stable TiO_2_ sol was obtained. The sol was spin coated at 3500 rpm for 30 s on a glass with diameter of 2 inches, which was ultrasonically cleaned for 10 min in a solution of acetone, 2-propanol and deionized water with volume ratios of 1:1:1. As-coated TiO_2_ precursor layer was heated under 400 °C for 1 h to produce fine grained TiO_2_ layer. The glass with TiO_2_ buffers was used for the hydrothermal growth of VO_2_ films. In the hydrothermal process, the vanadyl oxalate precursors were prepared by dissolving V_2_O_5_ (0.182 g) in the aqueous solution (50 ml) containing oxalic acid (1.97 g) at 70 °C. The aqueous solution was diluted into 500 ml with deionized water, forming a 4 mmol/L solution with pH value ~2.4. The PH value was controlled by NH_4_OH. The vanadyl oxalate aqueous solution (60 ml) was transferred into a Teflon-lined autoclave (100 ml). The chemical reaction was carried out at 230 °C in an electric oven. After heating for 4 h, the autoclave was naturally cooled down in furnace. The side of TiO_2_ layer was covered by a uniform film. The wafer samples were cleaned up with deionized water and alcohol, and dried by nitrogen. The thermochromic VO_2_ windows were obtained through annealing the as-grown VO_2_ films in a short annealing furnace at 400 °C for 60 s in 4 * 10^4^ Pa of air. Unless specifically noted in the article, all samples used here are prepared as mentioned above.

### Instrumentation characterization

The morphology of the reaction product was examined by using scanning electron micros-copy (SEM, Hitachi S-4800). The phase identification of the TiO_2_ and VO_2_ films was performed using X-ray diffraction (XRD, Bruker-AXS diffractometer, Model D8 ANVANCE) with Cu-Kα radiation source, Raman spectra (HR800, excitation wavelength: 633 nm, laser power: 1 mW) and Transmission Electron Microscope (TEM, FEI Tecnai G2 F20 S-TWIN). The chemical valence of vanadium ions was measured by XPS (PHI QUANTERA-II SXM) with Al-Kα radiation source (1486.6 eV). The porosity based on SEM images was calculated by using Image-Pro Plus (IPP) to compare the gray scale pixel of the area occupied by VO_2_ nanoplates and exposed TiO_2_ films. The optical transmittance spectra of samples at normal incidence from 300 to 3000 nm and were measured by using Shimadzu UV-3600 UV-VIS-NIR spectrophotometer with Heat Solid Transmission Accessory.

## Result and Discussion

**Figure 1:**
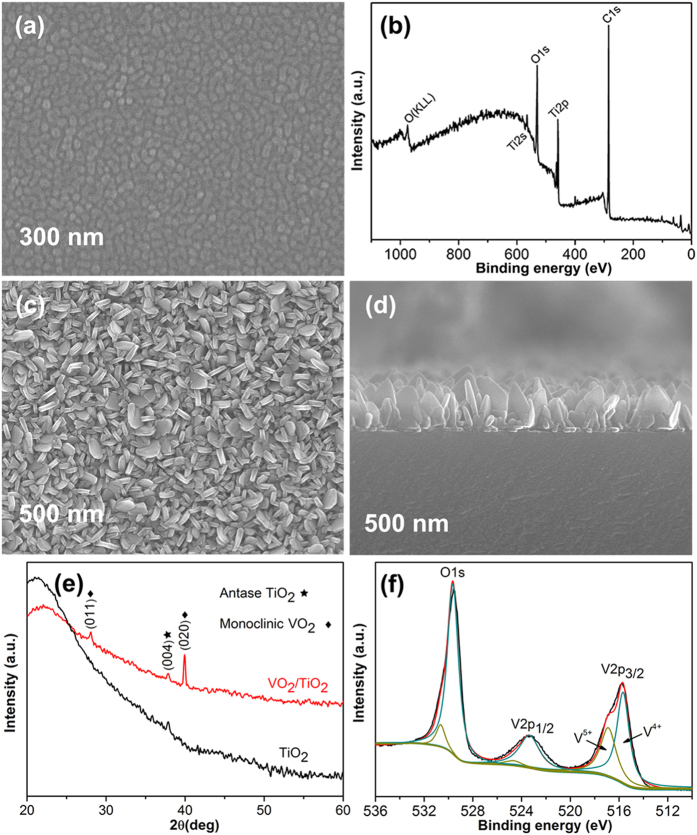
(**a**) Compact TiO_2_ thin films are composed of equiaxed grains with size distribution between 25 to 75 nm. (**b**) The XPS full spectrum of prepared TiO_2_ thin film. (**c**,**d**) SEM images of the obtained VO_2_ thin films and the corresponding cross section morphology, revealing a nanoplate structure. (**e**) XRD patterns of VO_2_ thin films compared to TiO_2_ thin films, indicating the orientated growth of the monoclinic VO_2_ on anatase TiO_2_ phase. (**f**) XPS spectrum of VO_2_ thin films.

**Figure 2:**
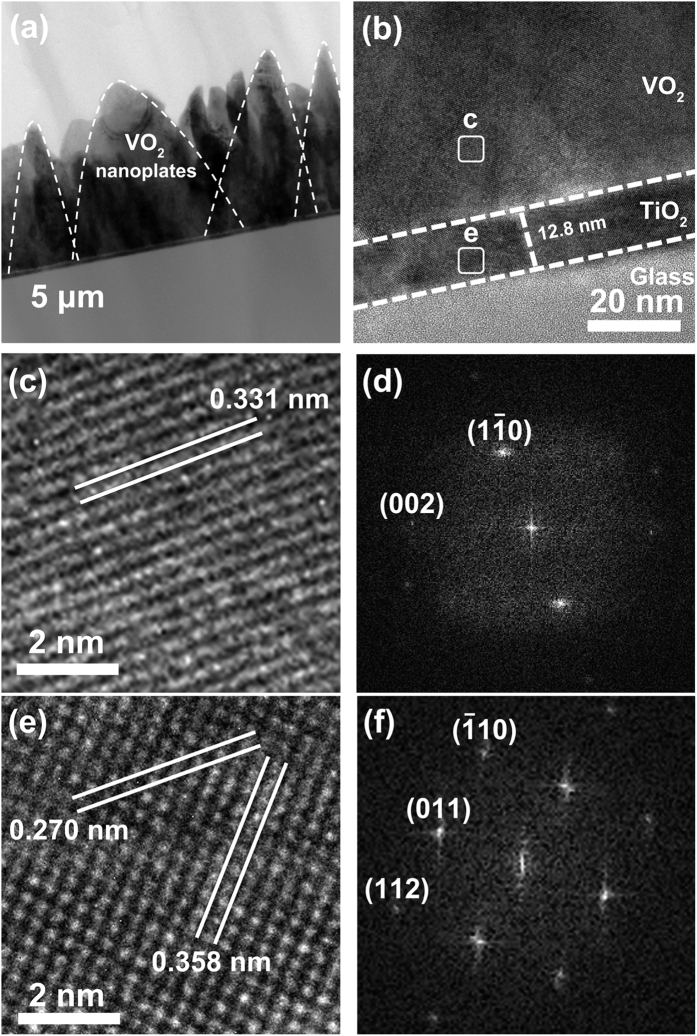
(**a**,**b**) Cross-sectional TEM images of the VO_2_/TiO_2_ films on glass substrate, (**a**) shows the shape of the VO_2_ nanoplates, (**b**) a VO_2_ grain grown on the TiO_2_ thin film, (**c**,**e**) High resolution TEM (HRTEM) images taken from different layers as marked by squares in (**b**). (**d**,**f**) FFT patterns correspond to (**c**) VO_2_ nanoplate and (**e**) TiO_2_ thin film respectively.

Figure 1a shows the morphology images of polycrystalline TiO_2_ buffers with grain size between 25 to 75 nm. The XPS full spectrum (Fig. 1b) of TiO_2_ reveals a high purity component. The obtained VO_2_ film is composed of nanoplates with an average thickness of ~40 nm, and a height of ~400 nm, which are regularly grown against substrates (Fig. 1c,d). There are smaller and more randomly oriented nanoplates close to the substrate, which is similar with the previous report for the growth of ZnO films^33^. As identified by XRD (Fig. 1e), the characteristic peaks agree with those of M-VO_2_ in monoclinic structure (JCPDS No. 65-2358) and A-TiO_2_ in anatase phase (JCPDS No. 21-1272) respectively. The remarkable (020) peak of VO_2_ indicates that the growth of VO_2_ films are preferentially oriented on substrates. For a randomly oriented VO_2_ polycrystalline sample the intensity of (020) diffraction is only ~2.4% of the strongest peak (011). The preferred orientation of the VO_2_ films supports the conclusion that the VO_2_ nanoplates are regularly grown on substrates as shown in the cross-section structure of VO_2_ films in Fig. 1d. The XRD pattern of TiO_2_ buffers indicates the (004)-preferred orientation of anatase TiO_2_. It is known that the close-packed planes in anatase-TiO_2_ (112) and rutile-VO_2_ (200)/(020) are equivalent^34^, so we can infer that there is a lattice-matching relationship between anatase TiO_2_ and rutile VO_2_ with *A*-TiO_2_ (112)//*R*-VO_2_ (200)/(020). In this case, it is possible for VO_2_ to grow in a preferred orientation manner guided by the *A*-TiO_2_ buffer under hydrothermal growth temperature (230 °C). The *M*-VO_2_ is a polymorphic phase transformed from *R*-VO_2_ through a small distortion^35^. The *R*-VO_2_ {200} planes correspond to the (020) and (002) planes in the *M*-VO_2_ phase^36^. For the (004)-preferred orientation of anatase TiO_2_ as determined by XRD, the preferred orientation of *M*-VO_2_ should be (011)*M* considering the crystal distortion induced by the mismatch between TiO_2_ and VO_2_. The angle between (112) and (004) in *A*-TiO_2_ is ~61° and no good lattice-match relation exist along other directions, therefore, the inclined growth of plate-like VO_2_ nanocrystals are observed in Fig. 1c,d. While the VO_2_ nanoplates show the strong preferred orientation of (020)*M*, it should be related to other orientations of TiO_2_, i.e. (110) or (112) orientations of *A*-TiO_2_. For *A*-TiO_2_ (110) or (112) orientations the VO_2_ nanoplates would grow perpendicular or parallel to the substrate. The corresponding growth of VO_2_ nanoplates can be observed in Fig. 1c,d. The existence of (110)-orientation TiO_2_ is verified by TEM in Fig. 2. XPS measurements were performed to examine the oxidation states of V ions in VO_2_ thin films (Fig. 1f)^37^. It is shown that the VO_2_ thin films contain partial V^5+^ ions together with V^4+^ ions. The presence of V^5+^ ions could be attributed to surface oxidization in the annealing process or storage in air and exist only on the surface as proved^5^.

In order to understand more details about the oriented growth of VO_2_ and TiO_2_ layers, a cross-section sample of VO_2_/TiO_2_ films was prepared and investigated by TEM. TEM images (Fig. 2a,b) show the well-connected 3-layer structure. The TiO_2_ thin film has a thickness ~12.8 nm (Fig. 2b). Two TiO_2_ grains exist in the observation region, and they have different orientations as shown by the HRTEM images in Fig. S1 (supporting information). The VO_2_ nanoplates show a triangle-like shape in Fig. 2a, which stand on the substrate. HRTEM images taken from two layers in Fig. 2c,e show clear lattice fringe, indicating good crystallinity of VO_2_ and TiO_2_ films. The interplanar spacing of 0.331 nm in Fig. 2c corresponds to the plane distance of (1–10) of monoclinic VO_2_ (Fig. 2d). The interplanar spacings of 0.270 nm and 0.358 nm in Fig. 2e belong to the (−110) plane and (011) plane of anatase TiO_2_ (Fig. 2f), respectively. For the present orientations of *A*-TiO_2_ and *M*-VO_2_ as shown in Fig. 2(c–f), the equivalent planes, i.e. *A*-TiO_2_ (112) and *M*-VO_2_ (002)/(020) are not in the matching orientations. However, the right-hand grain of *A*-TiO_2_ as shown in Fig. 2(b) and Fig. S1(c) exhibits an orientation that the left-hand grain rotates about 15° clockwise. In this case, the *M*-VO_2_ (002) plane is parallel to the *A*-TiO_2_ (112) plane of the right-hand grain, indicating the growth of VO_2_ in Fig. 2 is guided by the left-hand TiO_2_. The corresponding crystallographic relationship of VO_2_ and *A*-TiO_2_ is schematically shown in Fig. S2. The TEM analysis demonstrates the guided growth of VO_2_ by buffer TiO_2_.

To investigate the possible growth mechanism of VO_2_ films, controllable hydrothermal processes were designed. Different precursor solutions and pH values were found to be key factors to affect the reaction process. The role of precursors in the hydrothermal process for preparing the VO_2_ films were investigated, i.e. precursor solutions obtained from V(OH)_2_NH_2_ dissolved in HNO_3_ 38, hydrazine hydrate reacted with VOSO_4_ 39, NH_4_VO_3_ with 1,3-propylene glycol reduced in H_2_SO_4_ 40, and V_2_O_5_ dissolved in oxalate acid solution^41^. It is found that VO_2_ films can be grown only in the vanadyl oxalate solution, which suggests that oxalate acid solution is a suitable solvent for the formation of VO_2_ thin films.

**Figure 3:**
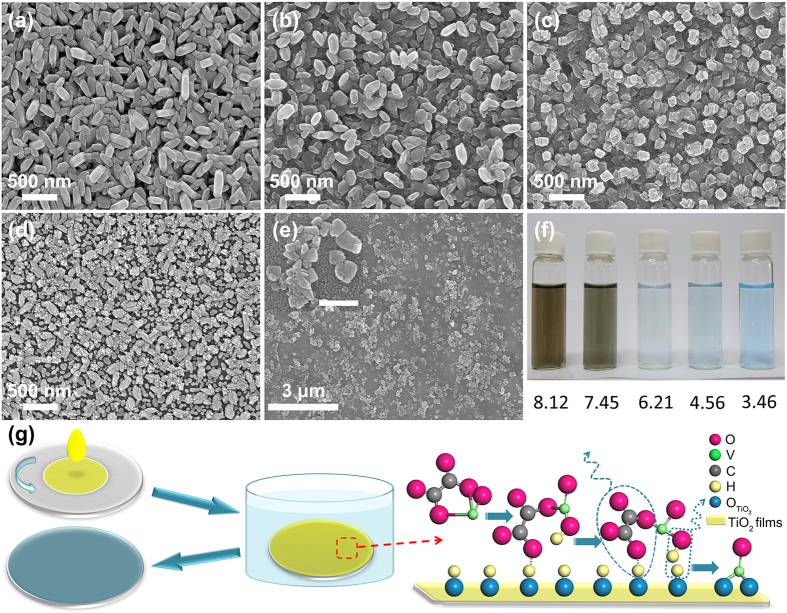
(**a**–**e**) SEM images of VO_2_ thin films grown in different PH value, (a, 3.46; b 4.56; c, 6.21; d, 7.45; e, 8.12). The image insert in (**e**) is high magnification, scale bar is 300 nm. (**f**) Photos of reaction solution with different PH value, the color of solution changes from light blue to dark brown reveals the vanadyl oxalate gradually decrease and eventually disappear. (**g**) Schematic illustration of process for fabricating VO_2_ film following an adsorption and dehydration process.

The pH value of vanadyl oxalate solution was modulated by adding droplets of NH_4_OH. Figure 3(a–e) show the SEM images of VO_2_ films prepared at different pH values. The morphology of VO_2_ nanoplates greatly changes with increasing pH values. Obviously, the growth of VO_2_ is greatly influenced by the pH value. At pH 3.46, the VO_2_ nanoplates in Fig. 3a are twice thicker than those grown at pH 2.4 (Fig. 1c), making the nanoplates more like nanorods (length was ~300 nm). When the pH value rises up to 4.56, the nanorods become shorter (length is 250 nm) and wider (Fig. 3b). As the PH value equals to 6.21, nanorods disappear instead of rectangle-like grains distribute randomly on the film (Fig. 3c). At pH 7.45, irregularly shaped particles are loosely attached to substrates. At PH 8.12, more area of substrate is exposed. Furthermore, experiments revealed that nothing could be grown on the substrate while pH values ≥ 8.5. Dobson *et al.* have examined the adsorption of low molecular weight (LMW) carboxylic acids to TiO_2_ in aqueous solutions by infrared spectroscopic analysis, and reported the existence of strong adsorption of dicarboxylic acids (such as oxalic acid) to TiO_2_ 42. This result was demonstrated by Mendive *et al.*, who pointed out that the pH value played an important role in the adsorption behavior^43^. The strong adsorption of oxalate organic species on TiO_2_ occurred only as the pH value less than IEP (the isoelectric point, a pH value at which a particular molecule or surface carries have no net electrical charge)^26^,^44^,^45^. Bandura *et al.* investigated the adsorption of H_2_O on TiO_2_, and reported that for adsorption of H_2_O onto the surface of TiO_2_, H^+^ and OH^−^ would produce positive (-O-H^+^) and negative (-Ti-OH^−^) surface sites, respectively^46^. The IEP of TiO_2_ is close to 6.2 as reported by Parks^47^. When PH is lower than 6.2, positive charge sites should dominate on the surface, whereas negative charge sites would be in majority. The adsorption affinity decreased rapidly as the pH value larger than IEP. Although the concentration of oxalate acids and the presence of metal cations in solution can influence the IEP, the pH dependence of adsorption does not change. It indicates that the protonated surface of TiO_2_ thin films is required for adsorption of organic anions. Our experimental results of different vanadic acid solutions and pH values are in consistence with the reported adsorption features of the organic acid solutions, indicating the chemical solution growth of VO_2_ on TiO_2_ is of adsorption dependence. The TiO_2_ buffer is a key factor for adsorption and consequently for interface reactions in the chemical solution environment because its surface chemical state at low pH values facilitates adsorption of carboxyl group.

In the oxalic acid solution, the possible surface reaction would be like that: 1) the vanadyl oxalate species were adsorbed on the TiO_2_ buffer. It is known that oxalate can form organic metallic cation complexes through the coordinating ability of the carboxyl group^48^. In that case, the negatively charged organic vanadium complexes ([(VO)_x_(C_2_O_4_)_y_]^x−y^) should be adsorbed on the positive surface sites through the carboxylic group. 2) Undergoing the water shrinkage reaction between the adsorbed vanadyl oxalate and the neighboring hydrogen ions on the protonated surface, VO^2+^ were adsorbed on the TiO_2_ substrate, and then crystallized to VO_2_ thin films. The schematic diagram of the growth process is shown in Fig. 3g. The different vanadic precursor solutions mentioned above have no carboxylic group, so there is no effective species to play the role of bridge between vanadium ions and positive charge-terminated surface of the TiO_2_ thin films for achieving the growth of highly adhesive VO_2_ films.

**Figure 4:**
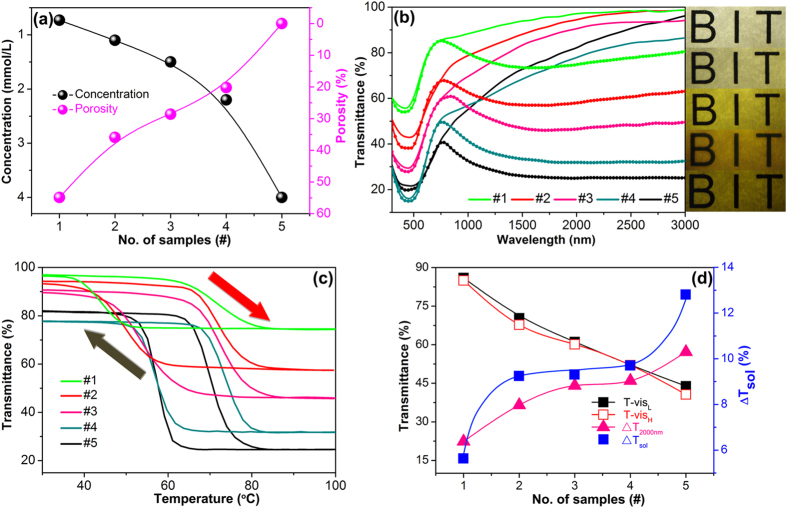
(**a**) Effect of concentration on the porosity of VO_2_ thin films, black and pink ball indicate the concentration and porosity respectively. (**b**) Transmittance spectra of different VO_2_ thin films from 300 nm to 3000 nm at 30 °C (solid dots line) and 100 °C (solid line), the right inserted photos from up to down are corresponding to the different samples: #1, #2, #3, #4, #5, respectively. (**c**) Thermal hysteresis loops of transmittance at 2000 nm for different VO_2_ thin films. The red arrow and black arrow indicate the heating and cooling respectively, and transition temperatures were defined as the center of the hysteresis loops. (**d**) Optical properties of typical samples with different precursor concentration. (The concentration of each sample as below: #1, 0.73 mmol/L; #2, 1.1 mmol/L; #3, 1.5 mmol/L; #4, 2.2 mmol/L; #5, 4.0 mmol/L, respectively).

The optical modulation properties of the prepared VO_2_ films were investigated to evaluate its potential for the smart windows. For realizing the application of VO_2_ in smart window a technological challenge is to improve the maximum visible transmittance (*T-vis*) to an acceptable value (>60%), while maintain the high solar modulating efficiency (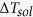) of VO_2_ 49. To improve *T-vis*, one way is to fabricate porous films^6^,^12^,^49^, and another way is to deposit an antireflection film or reduce the thickness of the continuous films of VO_2_ to less than 80 nm^12^,^24^. In this work, the standing nanoplate structure facilitates the penetration of solar light, namely apt to achieve high *T-vis*. The obtained VO_2_ films are in fact self-generated porous films, which would produce excellent combination thermochromic property. Cao *et al.* reported a nanoporous VO_2_ film exhibiting good thermochromic properties, the highest value of *T-vis* and 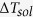 were 75% and 7.9% respectively^11^. In our work, the *T-vis* can be easily adjusted by changing the porosity of VO_2_ films through diluting the concentration of vanadyl oxalate in solution (Fig. S3). The porosity of VO_2_ films on glass increases with decreasing the concentration of vanadyl oxalate. By comparing the area occupied by VO_2_ nanoplates and exposed TiO_2_ films, the calculated porosities for the VO_2_ films grown in different concentration vanadyl oxalate solutions are shown in Fig. 4a. The samples are marked as: #1, 0.73 mmol/L; #2, 1.1 mmol/L; #3, 1.5 mmol/L; #4, 2.2 mmol/L; #5, 4.0 mmol/L (as in Fig. 1c), respectively. The sample #1 has the largest porosity of ~54.9%, it suggests that higher *T-vis* could be achieved.

Such self-generated porous nanostructures exhibit a good combination property of thermochromism (combining visible light transmittance and solar modulating efficiency). Figure 4b shows temperature-dependent transmittance of the porous VO_2_ nanoplates thin films. The right insets are the corresponding coating photos. The hysteresis loops of transmittance at 2000 nm for different VO_2_ thin films are shown in Fig. 4c, the *T*_*c*_ and hysteresis loop width (Δ*T*) of #5 is 70.1 °C and 12.9 °C respectively. Both of *T*_*c*_ and Δ*T* are increased as the porosity of thin films increasing, which is considered that the discontinuity of grain in thin films causes a loose grain boundaries limit propagation of MIT, and results higher *T*_*c*_ and wider Δ*T*^50^. The *T-vis*, 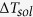, and near-infrared (NIR) switching efficiency (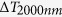) are shown in Fig. 4d, *T-vis* monotonously increases with the porosity of thin films as predicted. While the 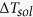 shows a plateau for samples #2-#4. Pleasurable thermochromic properties are observed in the sample #2 with 35.9% porosity, the *T-vis* value is as high as ~70.3% with the 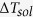 up to 9.3%. The results are even better than those of periodic and aperiodic porous VO_2_(M) films fabricated by complicated chemical and physical processes^6^,^24^,^49^, the multilayered TiO_2_(or SiO_2_)/VO_2_/substrate films^9^, and the VO_2_-based composite thin films^20^,^51^. The excellent thermochromic properties of our VO_2_ films benefit from the special nanoplates structure which provides pores to solve the issue of low visible transmittance, meanwhile keep the thickness of films up to ~400 nm.

The integrated solar transmittance (*T*_*sol*_, 300–2500 nm) and the 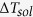 values are obtained from the following equation:


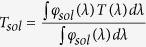



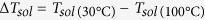


where *T*_*λ*_ denotes transmittance at wavelength *λ*, *φ*_*sol*_ is the solar irradiance spectrum for air mass 1.5 (corresponding to the sun standing 37° above the horizon)^52^.

## Conclusion

In summary, we have successfully fabricated nanoplates VO_2_ films on glass substrates with TiO_2_-buffers, for the first time, by a facile hydrothermal method. The obtained VO_2_ films show unique self-assembly porous structure with the porosity controllable by the concentration of the precursor solution. Excellent thermochromic properties are achieved with a visible light transmittance of 70.3% and a solar modulating efficiency of 9.3%. The investigation of growth process reveals that the appropriate adsorbent media, such as oxalate groups adsorbing on TiO_2_ buffers, are necessary for the preparation of VO_2_ thin films on glass by the hydrothermal technique. The preparation process of thermochromic VO_2_ films adopted in this work is facile, low-cost and up-scalable. The experiments proved its potential in promoting the practical application of VO_2_ in smart windows.

## Additional Information

**How to cite this article**: Zhang, J. *et al.* Hydrothermal growth of VO_2_ nanoplate thermochromic films on glass with high visible transmittance. *Sci. Rep.* **6**, 27898; doi: 10.1038/srep27898 (2016).

## Supplementary Material

Supplementary Information
