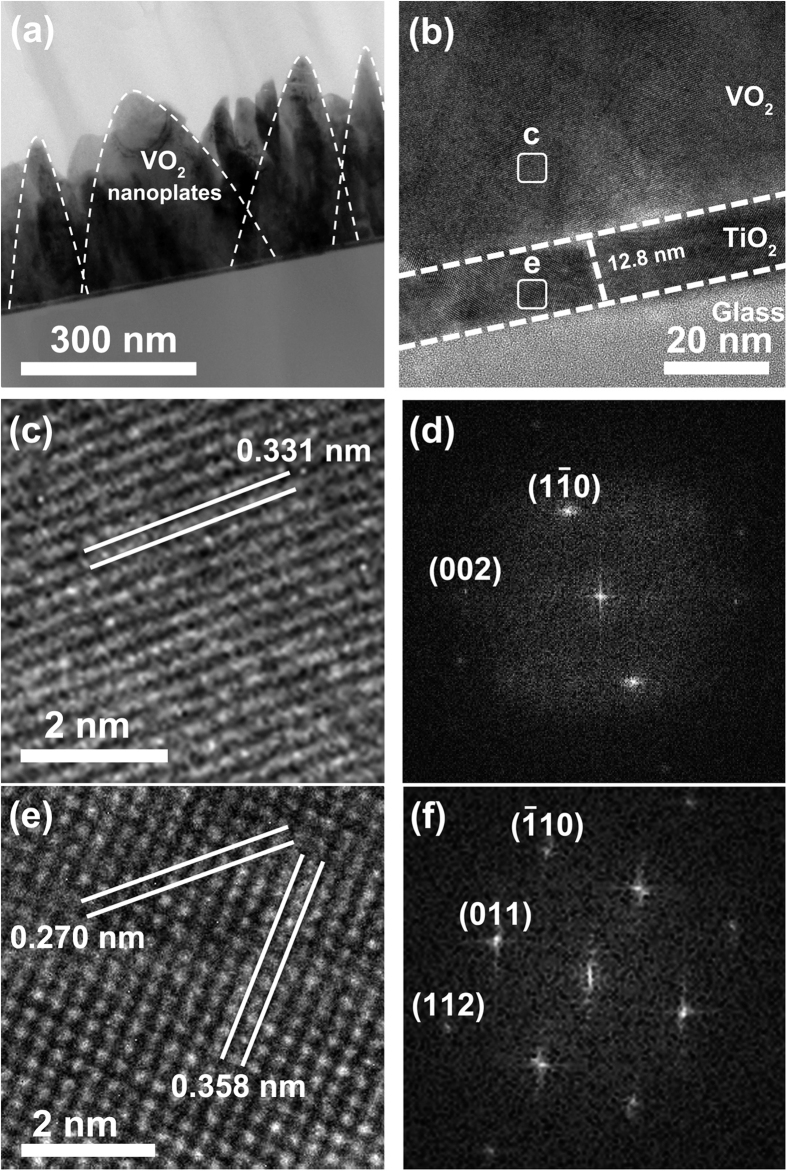# Corrigendum: Hydrothermal growth of VO_2_ nanoplate thermochromic films on glass with high visible transmittance

**DOI:** 10.1038/srep31018

**Published:** 2016-08-12

**Authors:** Jiasong Zhang, Jingbo Li, Pengwan Chen, Fida Rehman, Yijie Jiang, Maosheng Cao, Yongjie Zhao, Haibo Jin

Scientific Reports 6: Article number: 27898; 10.1038/srep27898 published online: 06 14 2016; updated: 08 12 2016.

In this Article, the scale bars are omitted from Figures 1a,c,d and 2a. The correct Figures 1 and 2 appear below.

## Figures and Tables

**Figure 1:**
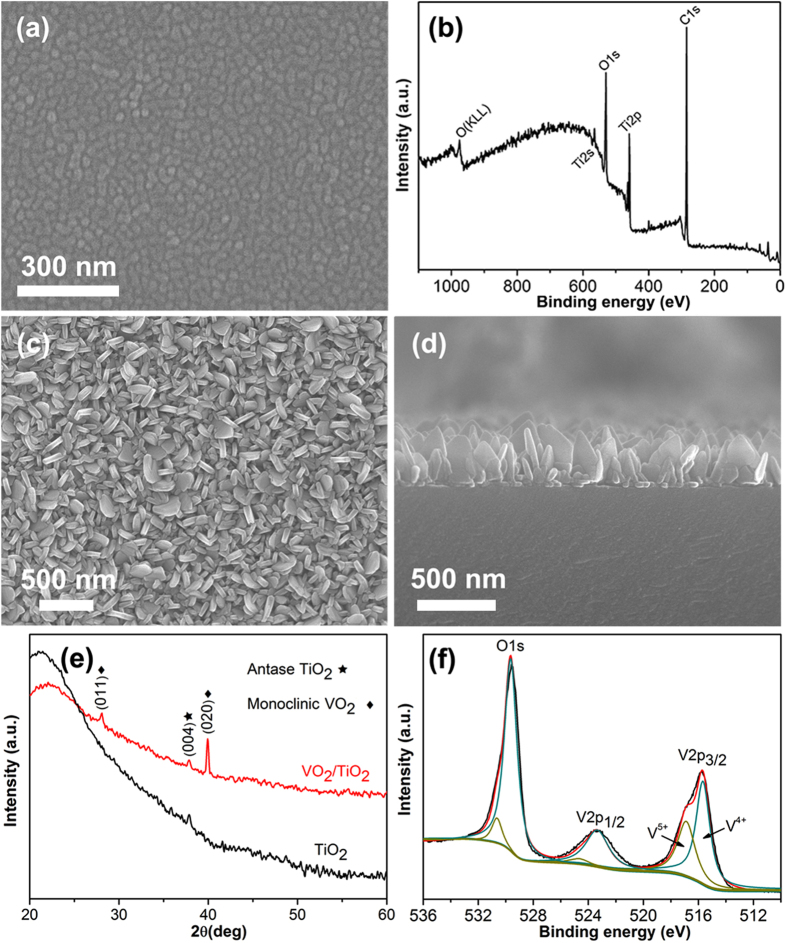


**Figure 2:**