# Supplementary material for: Hydrothermal growth of VO2 nanoplate thermochromic films on glass with high visible transmittance
Source: Sci Rep. 2016 Jun 14;6:27898. doi: 10.1038/srep27898 (PMC4906315; doi:10.1038/srep27898)
Supplement: Supplementary Information [file srep27898-s1.doc]

**Supplementary Information**

**Hydrothermal growth of VO_2_ nanoplate thermochromic films on glass with high visible transmittance**

Jiasong Zhang^1,2^, Jingbo Li^1,*^, Pengwan Chen^1^, Fida Rehman^1^,. Yijie Jiang^2^, Maosheng Cao^1^, Yongjie Zhao^1^, and Haibo Jin^1,*^

1. Beijing Key Laboratory of Construction Tailorable Advanced Functional Materials and Green Applications, School of Materials Science and Engineering, Beijing Institute of Technology, Beijing 100081, China.

2. Department of Mechanical Engineering and Applied Mechanics, University of Pennsylvania, Philadelphia, Pennsylvania 19104, USA.

*E-mail: lijb@bit.edu.cn; E-mail: [hbjin@bit.edu.cn](mailto:hbjin@bit.edu.cn).

KEYWORDS: VO_2_, Hydrothermal growth, Thermochromic property, Porosity


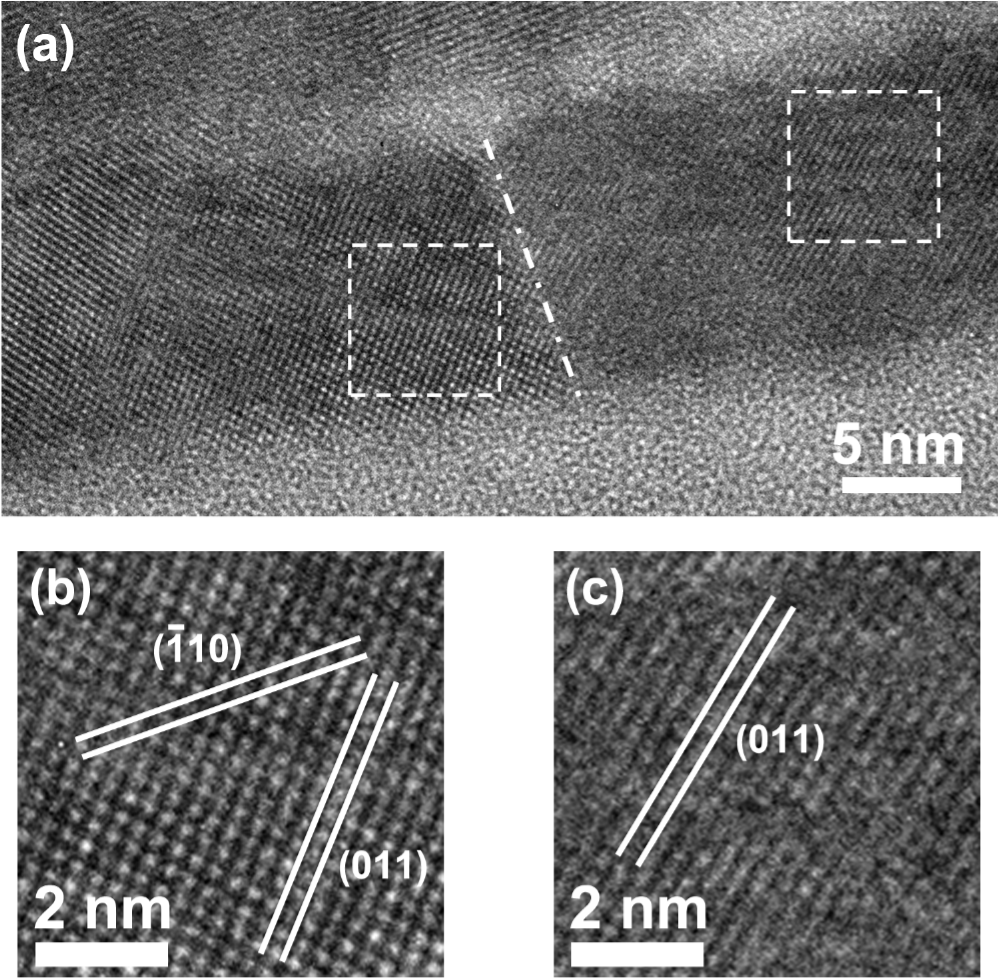


Figure S1. (a) Cross-sectional TEM image corresponding to the *A*-TiO_2_ buffer which shown in Figure 2b, revealing the different orientation of TiO_2_ grains, (b-c) high resolution TEM images taken from left-hand and right-hand grains in Fig. S1 (a) as marked by dashed square respectively.


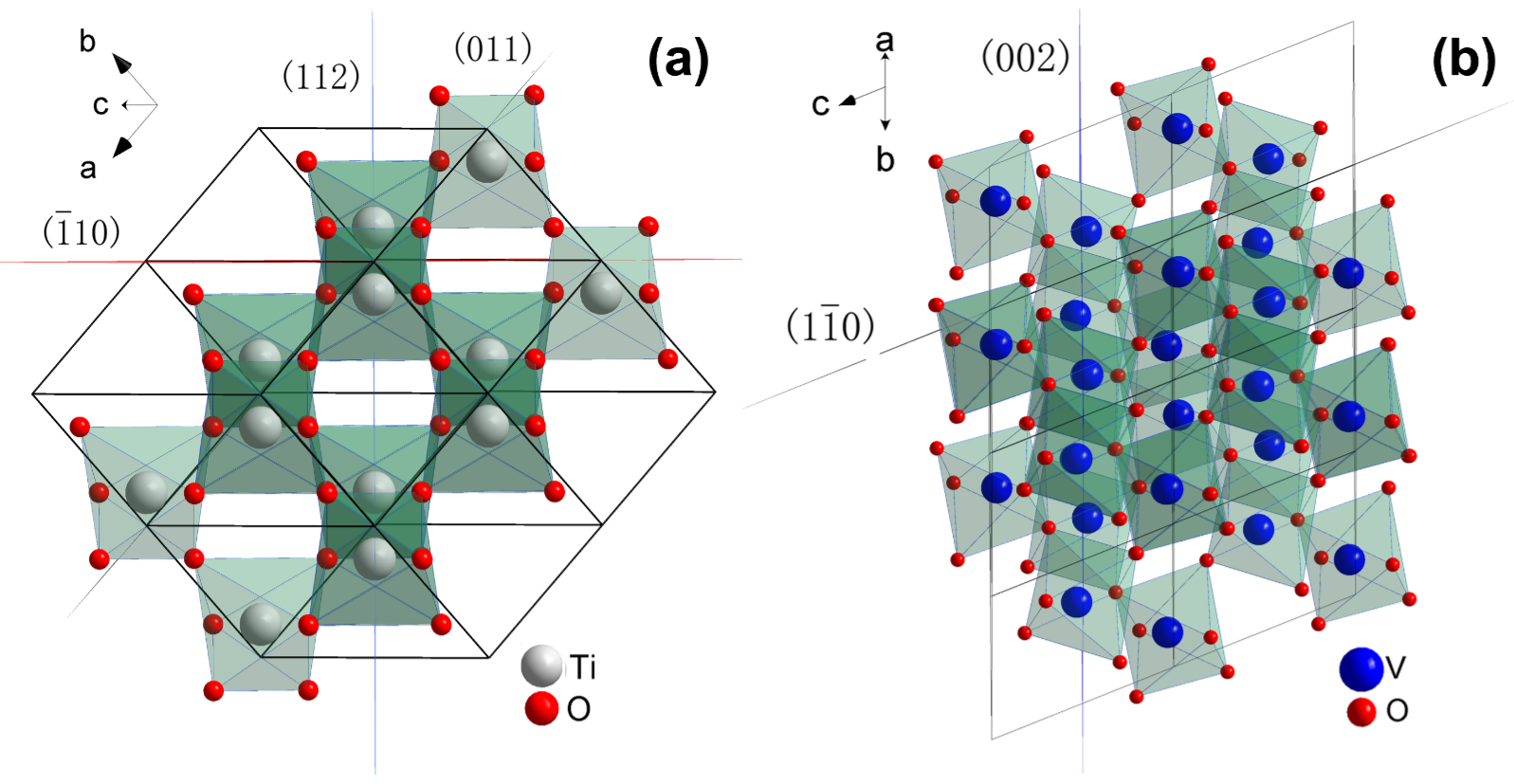


Figure S2. The schematic of crystallographic relationship of *M*-VO_2_ and *A*-TiO_2_, (a) corresponds to the right-hand TiO_2_ grain in Fig. S1(c); (b) corresponds to the VO_2_ grown on TiO_2_ in Fig. 2 and Fig. S1.


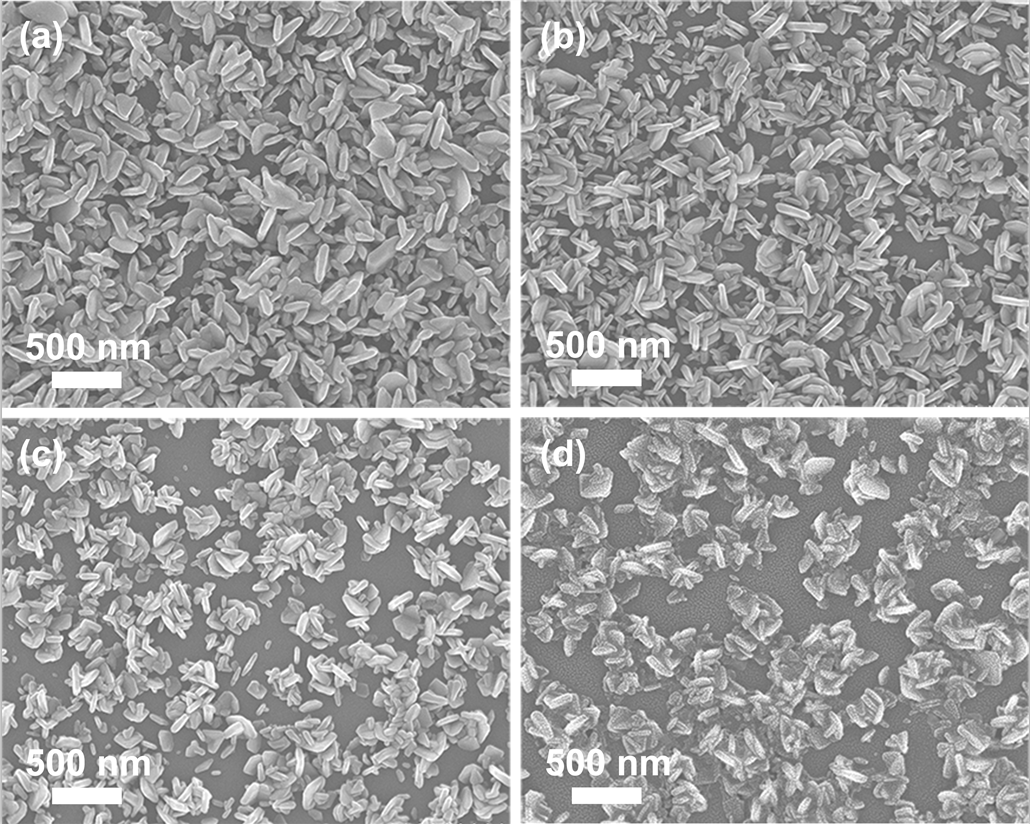


Figure S3. SEM images of the film grown on TiO_2_/glass substrate in different concentration vanadyl oxalate solution at 230 ^o^C for 4h.
